# Prevalence and Antimicrobial Resistance Pattern of *Salmonella*, *Shigella,* and Intestinal Parasites and Associated Factor among Food Handlers in Dilla University Student Cafeteria, Dilla, Ethiopia

**DOI:** 10.1155/2020/3150539

**Published:** 2020-05-27

**Authors:** Kuma Diriba, Ephrem Awulachew, Zemach Ashuro

**Affiliations:** ^1^Department of Medical Laboratory Sciences, Health Science and Medical College, Dilla University, Dilla, Ethiopia; ^2^School of Public Health, Health Science and Medical College, Dilla University, Dilla, Ethiopia

## Abstract

**Background:**

Food-borne disease is mostly caused by unsafe food handling and processing as well as poor hygienic practice. Recently, it is a worldwide and local burden to the human health. It is estimated that about one-third of the world population is affected by food-borne diseases annually and become a global public health problem. Hence, this study aimed to determine the prevalence, antimicrobial susceptibility patterns, and associated risk factors of *Shigella, Salmonella*, and intestinal parasites among food handlers in Dilla University, Southern Ethiopia.

**Methods:**

An institutional-based cross-sectional study was conducted from November to September 2018/2019. A structured questionnaire was used for the collection of data on sociodemographic characteristics. Parasite and bacterial identification, as well as susceptibility testing, was done using standard parasitological and bacteriological procedures.

**Results:**

Of the total 220 food handlers included in the study, 38.6%, 9.5%, and 3.2% were positive for intestinal parasites, *Salmonella*, and *Shigella species*, respectively. *A. lumbricoides* (11.4%) was the predominant parasite isolated followed by *E. histolytica* (7.7%). From the total *Salmonella* isolates, serogroup D was the most frequent isolate and from the total *Shigella* species, *Shigella flexneri* was the predominant isolate. In this study, through irregular medical checkups, those who drunk unpasteurized milk and ate raw meat were significantly associated with intestinal parasites. Both *Salmonella* and *Shigella* species were highly resistant to ampicillin (81%) and amoxicillin-clavulanic acid (>70%). *Salmonella* isolates are highly sensitive to cefotaxime and ceftriaxone, while *Shigella* is highly sensitive to ciprofloxacin and norfloxacin. MDR was recorded in 71.4% of all bacterial isolates.

**Conclusion:**

The presence of a high prevalence of intestinal parasites, *Salmonella,* and *Shigella* species that were resistant to the commonly prescribed drugs is a treat to the children and the community at large. Therefore, measures including health education, improvement of safe water supply, sanitation facilities, and continuous monitoring of microbiological and antimicrobial surveillance are crucial.

## 1. Introduction

Food-borne diseases are the most common disease mostly caused by unsafe food handling and processing as well as poor hygienic practice. Recently, it is a global and local burden to the community health. It is estimated that about one-third of the world population is affected by food-borne disease annually and two million deaths were reported each year. It is a public health problem in developed and developing countries [1]. The epidemiology of food-borne problems is complex and associated with the virulence factor of the organism and change of lifestyle, knowledge, poor hygienic practice, international travel, and migration and poverty [1, 2].

Both developed and underdeveloped countries are affected by intestinal parasitic infections throughout the world. Nearly one-third of the populations in developed countries are suffering from intestinal parasitic infection. This is around five times higher in developing countries [3]. In Ethiopia, intestinal parasitic infections are mainly associated with factors like poor socioeconomic conditions, poor hygiene and sanitation practices, unsafe and inadequate water supply, and environmental change [4]. The most common etiologic agents of intestinal parasitic infections in Ethiopia are *Ascaris Lumbricoides*, *Entamoeba histolytica*, *Giardia lamblia*, *Trichuris trichiura*, and *Hookworm* [5, 6].

Gastroenteritis is the most common health problem throughout the world which is caused by *Salmonella* and *Shigella* species [7, 8]. WHO reported about 16 million new cases and 600,000 deaths from typhoid fever each year throughout the world [8]. Diarrhea and dysentery are the most common life threatening diseases caused by *Shigella* strains called *Shigella dysentery* [9]. Due to the price and scarcity of laboratory facilities to perform accurate detection and antimicrobial susceptibility testing, the information relating to *Salmonella*, *Shigella* species, and their antimicrobial susceptibility patterns in Ethiopia is scarce. Thus, this study was aimed to determine the prevalence of *Salmonella* species, *Shigella* species, intestinal parasites, and their antimicrobial susceptibility patterns among food handlers working at Dilla University cafeterias.

## 2. Methods

### 2.1. Study Design

An institutional-based cross-sectional study was carried out among a total of 220 food handlers working at Dilla University, Southern Ethiopia, from November 2018 to September 2019. It found at a distance of 85 km from Hawassa and 365 km far from Addis Ababa. It is found in kola agroecological zone with an altitude of 1400 km above sea level and annual temperature ranging from 22°C to 29°C.

### 2.2. Sample Size and Sampling Technique

The sample size was calculated using a single population proportion formula by taking the prevalence of intestinal parasites (14.5%) conducted in Aksum, 5% margin of error (*d* = 0.05) and 95% confidence interval (*z* = 1.96). The initial sample size was 191, and by considering a 15% nonresponse rate, the final sample size was determined to be 220. To select representative participants, the final sample size was proportionally allocated to each stratum, and food handlers were selected using a systematic random sampling technique. Participants who reported to have never used any antimicrobial in the last 2 weeks and during the study period were included in the study.

### 2.3. Data Collection and Intestinal Parasite Identification

Data related to sociodemographic characteristics and personal hygiene practices were collected via a face to face interview method. After interviewing, respondents were asked to give a fresh stool specimen in a sterile, a clean wide-mouthed plastic container by clean wooden applicator stick, which was transported to microbiology laboratory for analysis. Two stool samples were collected; one stool sample will be immediately emulsified using saline (0.85% NaCl) for parasitological examination of trophozoite, cyst, larva, and ova stage. Direct wet mount preparation in normal saline, iodine solution, and formol-ether concentration sedimentation techniques were used for parasites identification.

### 2.4. Stool Culture

The isolation and characterization of *Salmonella* and *Shigella* species were performed based on the standard procedure [10]. Briefly, a mixture of a stool sample (1 mL) was transferred from the Cary Blair medium into a tube containing 9 mL of Selenite F broth (Oxoid, Ltd. UK) and incubated at 37°C for 24 hours to enrich the bacteria. An inoculum from Selenite F broth was subcultured on deoxycholate agar (DCA) and xylose lysine deoxycholate (XLD) agar (Oxoid, Ltd UK). After overnight incubation at 37°C the growth of *Salmonella* and *Shigella* was differentiated by their colony characteristic appearance on XLD agar (*Shigella*: red colonies, *Salmonella* red with a black center) and DCA (*Shigella*: pale colonies, *Salmonella* black center pale colonies). Pure colony with or without black centered on DCA or XLD were picked and suspended in sterile normal saline (0.85% NaCl) [11].

**Figure 1:**
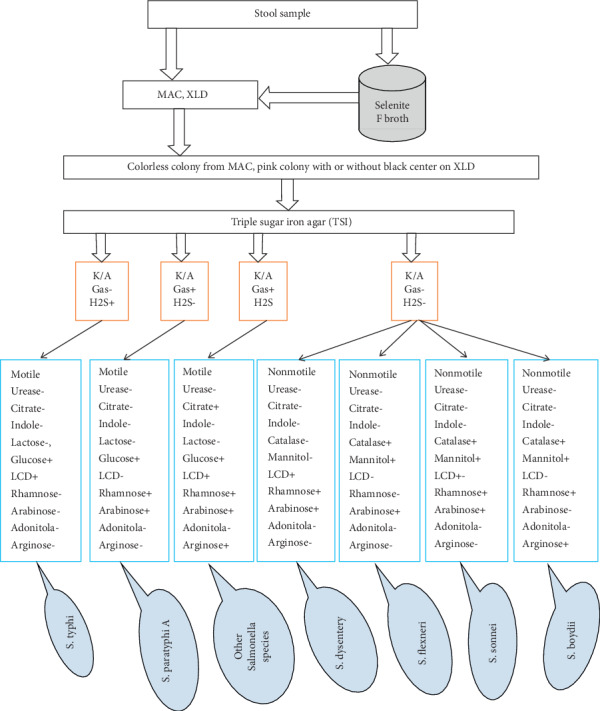
Flow chart for identification of *Salmonella* and *Shigella* species from stool sample.

Further identification was done biochemically using analytical profile index 20E (API 20E; bioMérieux SA, France). API 20E microtubes were filled up to the edge with the suspension. Sterile oil was added into the rhamnose, arabinose, arginine, adonitol, mannitol, ornithine decarboxylase, hydrogen sulfide, and urease production test compartments to create anaerobiosis [12, 13]. Reading of the result was done as per the manufacturer's instruction. *Salmonella* serogrouping was done by slide agglutination technique using poly O (AI) and monovalent (O2, O3, O4, O5, O6, O7, O8, O9, O15, and Vi) antigens for identification of *Salmonella* serogroups, A–E [14, 15]. (Figure 1).

### 2.5. Antimicrobial Susceptibility

Antimicrobial susceptibility tests were performed for each identified bacterium by disc diffusion method on Mueller Hinton Agar (Oxoid, Hampshire, UK) based on EUCAST guidelines [16]. The following antimicrobial agents, all from Oxoid, were used: ampicillin (10 *μ*g), amoxicillin-clavulanic acid (30 *μ*g), cefotaxime (5 *μ*g), ceftazidime (10 *μ*g), ceftriaxone (30 *μ*g), cefuroxime (30 *μ*g), chloramphenicol (30 *μ*g), ciprofloxacin (5 *μ*g), norfloxacin (10 *μ*g), and trimethoprim sulfamethoxazole (1.25/23.75 *μ*g). The resistance and sensitivity results were interpreted according to the clinical and laboratory standards institute [17]. MDR was defined as acquired nonsusceptibility to at least one agent in three or more antimicrobial categories.

### 2.6. Statistical Analysis

Data were edited, cleaned, entered, and analyzed using the statistical package for social science (SPSS) version 22. Descriptive analysis such as frequency and mean was used. Initially, the association between each exposure and the presence of infection was assessed using multivariate logistic regression and chi-square test, and odds ratios were computed to measure the strength of the association. *P* value of <0.05 was considered to indicate statistically significant differences.

### 2.7. Ethical Consideration

Ethical clearance was obtained from the Ethical Review Committee of Dilla University medical and health Science College. Written informed consent was obtained from each study participant. Strict confidentiality was maintained during the interview process and anonymity was kept during data processing and report writing. Food handlers who have found to be positive for enteric pathogens (parasite and bacterial) were referred to their respective staff medical center for appropriate antiparasitic and antimicrobial treatments.

## 3. Result

### 3.1. Sociodemographic Characteristics

A total of 220 food handlers were included in this study. Of these, 90.9% were females. The majority of the study subjects were between the age group of 20 and 40 years (84.5%) with the mean age of 31.2 (standard deviation ± 8.8 years). Most of the study participants had completed secondary school (61%). 56.4% of the study participants were married. Most of the study participants had served in the cafeteria for more than 5 years (77.3%). Only study participants with educational status of elementary and secondary schools and food handlers serving in the cafeteria for <5 years were significantly associated with intestinal parasites (*P* value <0.05) (Table 1).

### 3.2. Food Preparation and Personal Hygiene Related Factors of Food Handlers

Of the total respondents, 51.4% reported that they always perform periodic medical checkups. Similarly, 79.5% reported that they always use soap and water for hand washing after visiting toilet. The finding from observation also supported that personal hygiene was practiced by the food handlers. From the total, 86.8% of the study participants had drunk unpasteurized milk and only 42.3% eat raw meat. Only study participants who did not perform a periodical medical checkup, those who drink unpasteurized milk, and those who were eating raw meat were significantly associated with intestinal parasites (*P* value <0.007) (Table 2).

### 3.3. Prevalence and Types of Intestinal Parasite

The overall prevalence of intestinal parasites was 38.6% with eight different species. *Ascaris lumbricoides* was the leading parasite isolated (11.4%), followed by *Entamoeba histolytica* (7.7%) and *Giardia lamblia* (6.4%). The least parasites isolated were *Enterobius vermicularis* (0.9%) and *Hookworm* (0.5%). No double parasites were isolated from the study participant (Table 3).

### 3.4. Prevalence of Salmonellosis and Shigellosis

The overall prevalence of *Salmonella* and *Shigella* species was 12.7%. Of these, *Salmonella* and *Shigella* species each account for 9.5% and 3.2%, respectively. From the total *Salmonella* species, serogroup D was predominantly isolated with a prevalence of 52.4%, followed by serogroups C (33.3%) and B (14.3%). From the total *Shigella* species by biochemical identification. *Shigella flexneri* was predominantly isolated with the prevalence of 57.1%, followed by *Shigella boydii* with the prevalence of 28.6%.

### 3.5. Antimicrobial Resistance Pattern of the Isolated *Salmonella* and *Shigella* species

Antimicrobial susceptibility tests showed that *Salmonella* isolates were highly resistant to ampicillin (81%), and amoxicillin-clavulanic acid and chloramphenicol each accounting for (71.4%). More than 85% of *Salmonella* isolates were sensitive to cefotaxime and ceftriaxone. Among the *Salmonella* isolates, serogroup D showed a higher resistance rate to ampicillin (81.8%) and amoxicillin-clavulanic acid (81.8%) and serogroup C showed higher resistance to ampicillin (85.7%). All *Shigella* isolates showed 85.7% resistance to ampicillin, cefuroxime, and amoxicillin-clavulanic acid. More than 85% of *Salmonella* isolates were sensitive to ciprofloxacin and norfloxacin (Table 4).

### 3.6. Antibiogram Pattern of Multidrug Resistant *Salmonella* and *Shigella* species

Among the total isolates, multidrug resistance (MDR) was recorded in 71.4%. In this study, 66.7% of *Salmonella* isolates and 85.7% of *Shigella* isolates were recorded as MDR. Among *Salmonella* species, serogroup D (72.7%) followed by serogroup B (66.7%) showed a high level of MDR, while among *Shigella* species, *S. sonnei* and *S. boydii* showed100% level of multidrug resistance (Table 5).

### 3.7. Factors Associated with Intestinal Parasitosis among Food Handlers

Multivariable logistic regression results showed that food handlers who served less than 5 years in the cafeteria, food handlers who did not attend periodic medical checkup, food handlers who drink unpasteurized milk, and food handlers who ate raw meat have shown significant association (*P* < 0.05) (Table 6).

## 4. Discussion

In this study, a total of 220 food handlers were included. The prevalence of parasitic infection reported in the current study was 38.6%. This is consistent with studies done in Bahirdar [18], in Nigeria [19], and in India [20] with prevalence of 41.1%, 38.1%, and 29.3%, respectively; but it is higher than the studies conducted in Iran [21] (11.9%), in Sudan [22] (6.9%), and in Ghana [23] (21.6%). However, it is lower as compared to the findings reported in Mekele [24] (49.3%) and in Anatolia [25] (52.2%). The differences might be due to variation in years of study, sociodemographic characteristics, personal hygiene practice and environmental sanitation, safe water supply, health promotion practice, food hygiene and safety training, knowledge of transmission, and prevention of intestinal parasite differences.

In the current study, *Ascaris lumbricoides* (11.4%) was the predominant parasite identified followed by *Entamoeba histolytica* (7.7%) *and Giardia lamblia* (6.4%). Similar findings have been reported in previous studies done in Ethiopia [26–29]. Studies done in Aksum town [6] (5%), in Iran [21] (3.7%), and Saudi Arabia [30] (9%) reported *Giardia lamblia* as the predominant intestinal parasite, but the study conducted in Addis Ababa [29] (70.8%) reported *Entamoeba histolytica* as the predominant parasite. The high prevalence of those parasites might be due to low personal hygiene practice and the easy mode of transmission of the parasite which is usually found in unsafe food, water, soil, or contaminated surface with feces.

The finding of this study showed that the odds of being positive for intestinal parasitic infection is higher among food handlers who did not attend periodic medical checkup compared to those who had attended periodic medical checkup. This figure is inconsistent with a report from Aksum [6], Mekelle [24], and Jimma Town [31]. In the current study, Food handlers who drink pasteurized milk were 81% less likely to be positive for intestinal parasites compared to those who drink unpasteurized milk. Food handlers who did not eat raw meat were 97% less likely to be positive for intestinal parasites compared to those who ate raw meat. This is to mean that most of the intestinal parasitosis was transmitted by eating raw meat and drinking unpasteurized milk.

In this study, the prevalence of *Salmonella* species was 9.5% which is in line with the finding reported in Arbaminch [32] (6.9%) and in Sodo town [33] (8.8%) but higher than the study conducted in different area of Ethiopia [29, 34–36] with prevalence ranging from 1.6% to 5%. On the contrary to our study, higher findings were reported in Ethiopia [37] and Nigeria [19] with a prevalence of 13.5% and 31.5%, respectively. The differences may be due to the environmental condition, study area, and the laboratory method used for the bacterial identification.

In the current study, serogroup D was predominantly isolated with a prevalence of 52.4% followed by serogroup C (33.3%). This is in line with a study conducted in different areas of Ethiopia where serotypes D, C, and B were the leading groups interchangeably [38–41]. On the other hand, serogroup C occurred more frequently than serogroups D and B in Central and North Ethiopia [39, 42]. The difference might be due to differences in study period, the study area, and differences in environmental conditions.

*Salmonella typhi* as a common etiologic agent of gastroenteritis and typhoid fever is a public health concern as it was evidenced in Southern Ethiopia to be the predominant illness among food handlers [43]. In this study, the prevalence *S. typhi* was 3.1% which agrees with studies conducted in India [7], in Sodo town [33], and in Bahirdar [18] with prevalence of 3.8%, 2.6%, and 2.7%, respectively, but higher findings were reported in Hawassa University [33] (8.1%) and in Jordan [44] (17.4%). However, lower finding was reported in in Gondar University [45] (1.3%). Differences in the prevalence of *S. typhi* could be attributed to differences in lifestyle and diagnostic technique and differences in study area and recent or previously treated infection. The lower living standard and poor hygienic matters of the general population are suggestive evidence that enteric fever is a threat in present-day Ethiopia.

The isolation rate of *Shigella* species in this study was 3.2%, which may indicate food handlers' low hygienic status and may lead to outbreaks of bacillary dysentery among the student population. This finding is consistent with the study conducted in Arba Minch University [32] (3%) and Gondar [45] (2.7–3.1%), however, lower than the findings of other studies conducted in Nigeria [19] (15.5%), but the present result was higher than the reports in Abeokuta [46] (0%). The discrepancy may be due to the difference in a technique of pathogens isolation, type of study participant, lifestyle, and sample size.

The resistance rates for the isolated *Salmonella* species in this study were high for ampicillin, amoxicillin, and chloramphenicol (>71%). Our finding was comparable with previous studies conducted in Sodo town [33], Gondar [45], Haramaya [47], and Central Ethiopia [48]. Cefotaxime and ceftriaxone resistant *Salmonella* isolates were not revealed in previous studies in Ethiopia [48, 49] in contrary to our study. This may indicate the emerging of ceftriaxone and cefotaxime resistance isolates over time. *Shigella* species were highly resistant to ampicillin, amoxicillin, and cefuroxime. This was consistent with the study done in another region of Ethiopia [32, 45, 47, 50]. The magnitude of MDR *Salmonella* and *Shigella* species in this study was 66.7% and 85.7%, respectively, which is comparable with the previous findings in Ethiopia [33] but lower than 100% resistance in Addis Ababa University [29]. The high MDR rate of *Salmonella* and *Shigella* isolates for most of the antibiotics currently used could limit our antibiotic option for empirical therapy.

The current study indicates a higher prevalence of *Salmonella* and *Shigella* species among food handlers who had between 1 and 5 years' work experience (71.5%) compared to less than 1 year (10.2%) or greater than 5 years (18.3%). This is in line with a study done in Mekelle University [51] in which 60% of food handlers who served for less than 5 years were infected. However, our finding is lower than that of the study done in Arba Minch University [32], where 32.4% of food handlers who served for greater than 5 years were infected. Lack of regular medical checkups, food safety training, inadequate supervision, and a low level of literacy of food handlers might contribute to this difference.

## 5. Conclusion

In this study, 38.6% of stool specimens were positive for different intestinal parasites. *Salmonella* isolation rate was 9.5%, of which >71% were resistant to ampicillin, amoxicillin-clavulanic acid, and chloramphenicol and >85% sensitive to cefotaxime and ceftriaxone. *Shigella* isolation rate was 3.2%, of which >85% were resistant to ampicillin, cefuroxime, and amoxicillin-clavulanic acid and showed the least resistance to ciprofloxacin and norfloxacin. Serogroup D among *Salmonella* species, *Shigella flexneri* among *Shigella species*, and *A. lumbricoides* among intestinal parasites were the predominant isolates. Among the total isolates, multidrug resistance was recorded in 71.4% of all bacterial isolates. Of the checked risk factors, irregular medical checkups, drinking unpasteurized milk, and eating raw meat were significantly associated with pathogens infection. Therefore, constant epidemiological surveillance, improvement of personal hygiene, and drinking pasteurized milk and eating cooked meat are recommended to control pathogens infection in food handlers.

## Figures and Tables

**Table 1:** Sociodemographic characteristics of food handlers in Dilla University students' cafeteria, Southern Ethiopia (November 2018–September 2019) (*n* = 220).

Variables		Intestinal parasite isolated				*P* value
		Positive	Percent	Negative	Percent	
Sex	Male	5	25	15	75	0.589
	Female	60	30	140	70	1
Age in year	<20	5	50	5	50	0.146
	20–40	50	26.9	136	73.1	0.478
	>40	10	41.7	14	58.3	1
Educational status	Elementary school	2	10	18	90	0.046
	Secondary school	49	36.6	85	63.4	0.034
	Collage and above	14	21.2	52	78.8	1
Monthly income in ETB	<2000	29	30.5	66	69.5	0.998
	>2000	36	28.8	89	71.2	1
Family size	<2	27	27.6	71	72.4	0.911
	2–5	27	32.1	57	67.9	0.888
	>5	11	28.9	27	71.1	1
Marital status	Single	30	31.9	64	68.1	0.943
	Married	34	27.4	90	72.6	0.731
	Divorced	1	50	1	50	1
Service year	<1 year	2	16.7	10	83.3	0.038
	1–5 years	21	55.3	17	44.7	0.003^*∗*^
	>5 years	42	24.7	128	75.3	1

**Table 2:** Food preparation and personal hygiene related factors of food handlers in Dilla University students' cafeteria, Southern Ethiopia (November 2018–September 2019) (*n* = 220).

Variables		Intestinal parasite isolated				*P* value
		Positive	Percent	Negative	Percent	
Job position	Preparation	41	33.6	81	66.4	0.113
	Cleaning utensil	5	31.2	11	68.8	0.200
	Host students	19	23.2	63	76.8	1
Medical checkups	Yes	1	0.9	112	99.1	<0.001^*∗*^
	No	64	59.8	43	40.2	1
Hands washing habit	Yes	64	29.6	152	70.4	0.185
	No	1	25	3	75	1
Finger nail status	Trimmed	54	30	126	70	0.353
	Not trimmed	11	27.5	29	72.5	1
Wear clean apron	Yes	43	30.9	96	69.1	0.345
	No	22	27.2	59	72.8	1
Wear hair garment	Yes	45	29.6	107	70.4	0.320
	No	20	29.4	48	70.6	1
Knowledge about salmonellosis	Yes	20	29	49	71	0.727
	No	45	29.8	106	70.2	1
Hands wash with soap	Only with water	12	26.7	33	73.3	0.673
	With soap	53	30.3	122	69.7	1
Source of water for drinking	Pipe	61	28.5	153	71.5	0.677
	Hand dug well	3	100	0	0	0.377
	Other	1	33.3	2	66.7	1
Unpasteurized milk	Yes	15	51.7	14	48.3	0.007^*∗*^
	No	50	26.2	141	73.803.	1
Eating raw meat	Yes	61	65.6	32	34.4	<0.001^*∗*^
	No	4	3.1	123	96.9	1
Eating uncooked vegetable	Yes	24	27.6	63	72.4	0.230
	No	41	30.8	92	69.2	1

**Table 3:** Prevalence of intestinal parasites detected from stool specimens of food handlers in Dilla University students' cafeteria, Southern Ethiopia (November 2018–September 2019) (*n* = 220).

Sr. no.	Type of parasites	Frequency	Percentage (%)
1	*Ascaris lumbricoides*	25	11.4
2	*Entamoeba histolytica*	17	7.7
3	*Giardia lamblia*	14	6.4
4	*Taenia* species	13	5.9
5	*Trichuris trichiura*	9	4.1
6	*Hymenolepis nana*	4	1.8
7	*Enterobius vermicularis*	2	0.9
8	*Hookworm*	1	0.5

**Table 4:** Antimicrobial resistance patterns of *Salmonella* and *Shigella* species isolated from food handlers in Dilla University student cafeteria, Southern Ethiopia (November 2018–September 2019) (*n* = 220).

Antibiotics	Pattern	Antimicrobial susceptibility test of *Salmonella* species			Antimicrobial susceptibility test of *Shigella* species		
		Serogroup B (*n* = 3)	Serogroup C (*n* = 7)	Serogroup D (*n* = 11)	*S. flexneri* (*n* = 4)	*S. sonnei* (*n* = 1)	*S. boydii* (*n* = 2)
**AMP**	S	1 (33.3%)	1 (14.3%)	2 (18.2%)	1 (25%)	0 (0.0%)	0 (0.0%)
	R	2 (66.7%)	6 (85.7%)	9 (81.8%)	3 (75%)	1 (100%)	2 (100%)
**AMX**	S	1 (33.3%)	3 (42.9%)	2 (18.2%)	1 (25%)	0 (0.0%)	0 (0.0%)
	R	2 (66.7%)	4 (57.1)	9 (81.8%)	3 (75%)	1 (100%)	2 (100%)
**CIP**	S	2 (66.7%)	5 (71.4%)	9 (81.8%)	4 (100%)	1 (100%)	1 (50%)
	R	1 (33.3%)	2 (28.6%)	2 (18.2%)	0 (0.0%)	0 (0.0%)	1 (50%)
**CRO**	S	2 (66.7%)	6 (85.7%)	10 (90.9%)	2 (50%)	1 (100%)	1 (50%)
	R	1 (33.3%)	1 (14.3%)	1 (9.1%)	2 (50%)	0 (0.0%)	1 (50%)
**C**	S	1 (33.3%)	2 (28.6%)	3 (27.3%)	2 (50%)	0 (0.0%)	1 (50%)
	R	2 (66.7%)	5 (71.4%)	8 (72.7%)	2 (50%)	1 (100%)	1 (50%)
NOR	S	2 (66.7%)	6 (85.7%)	9 (81.8%)	3 (75%)	1 (100%)	2 (100%)
	R	1 (33.3%)	1 (14.3%)	2 (18.2%)	1 (25%)	0 (0.0%)	0 (0.0%)
**CEFO**	S	3 (100%)	6 (85.7%)	10 (90.9%)	3 (75%)	1 (100%)	1 (50%)
	R	0 (0.0%)	1 (14.3%)	1 (9.1%)	1 (25%)	0 (0.0%)	1 (50%)
**CEFU**	S	2 (66.7%)	6 (85.7%)	9 (81.8%)	1 (25%)	0 (0.0%)	0 (0.0%)
	R	1 (33.3%)	1 (14.3%)	2 (18.2%)	3 (75%)	1 (100%)	2 (100%)
**CEZ**	S	2 (66.7%)	6 (85.7%)	9 (81.8%)	2 (50%)	0 (0.0%)	1 (50%)
	R	1 (33.3%)	1 (14.3%)	2 (18.2%)	2 (50%)	1 (100%)	1 (50%)
**SXT**	S	1 (33.3%)	4 (57.1%)	6 (54.5%)	2 (50%)	0 (0.0%)	1 (50%)
	R	2 (66.7%)	3 (42.9%)	5 (45.5%)	2 (50%)	1 (100%)	1 (50%)

AMP = ampicillin, AMX = amoxicillin-clavulanic acid, CEFO = cefotaxime, CEZ = ceftazidime, CRO = ceftriaxone, CEFU = cefuroxime, C = chloramphenicol, CIP = ciprofloxacin, NOR = norfloxacin, and SXT = trimethoprim sulfamethoxazole.

**Table 5:** Multidrug resistance pattern of *Salmonella* and *Shigella* species isolated from food handlers in Dilla University student cafeteria, Southern Ethiopia (November 2018–September 2019) (*n* = 220).

Multidrug resistance	Multidrug resistance *Salmonella* species			Multidrug resistance *Shigella* species		
	Serogroup B (*n* = 3)	Serogroup C (*n* = 7)	Serogroup D (*n* = 11)	*S. flexneri* (*n* = 4)	*S. sonnei* (*n* = 1)	*S. boydii* (*n* = 2)
R0	0 (0.0%)	1 (14.3%)	1 (9.1%)	0 (0.0%)	0 (0.0%)	0 (0.0%)
R1	0 (0.0%)	1 (14.3%)	0 (0.0%)	0 (0.0%)	0 (0.0%)	0 (0.0%)
R2	1 (33.3%)	0 (0.0%)	2 (18.2%)	1 (25%)	0 (0.0%)	0 (0.0%)
R3	1 (33.3%)	2 (28.6%)	2 (18.2%)	2 (50%)	1 (100%)	1 (50%)
R4	1 (33.3%)	1 (14.3%)	1 (9.1%)	0 (0.0%)	0 (0.0%)	0 (0.0%)
R5	0 (0.0%)	1 (14.3%)	3 (27.3%)	1 (25%)	0 (0.0%)	1 (50%)
R6	0 (0.0%)	0 (0.0%)	1 (9.1%)	0 (0.0%)	0 (0.0%)	0 (0.0%)
≥R7	0 (0.0%)	0 (0.0%)	1 (9.1%)	0 (0.0%)	0 (0.0%)	0 (0.0%)

Ro = bacterial isolate sensitive to all antibiotics, R1, R2, R3, R4, R5, R6, and ≥R7 = bacterial isolate resistance to 1, 2, 3, 4, 5, 6, and ≥7 antibiotics, respectively.

**Table 6:** Multivariable logistic regression analysis of predicators for intestinal parasitic infection among food handlers in Dilla University student cafeteria, Southern Ethiopia (November 2018–September 2019), (*n* = 220).

Variables		Positive for intestinal parasite		COR (95% CI)	*P* value	AOR (95% CI)
		Yes	No			
Sex	Male	5	15	1.29 (1.44–3.70)	0.589	2.43 (0.31–18.93)
	Female	60	140	1		1
Educational status	Elementary school	2	18	2.42 (1.50–11.71)	0.046	1.13 (1.25–5.05)
	Secondary school	49	85	0.48 (1.23–1.93)	0.034	0.95 (1.13–1.86)
	Collage and above	14	52	1		1
Service year	<1 year	2	10	0.27 (0.13–0.56)	0.038	0.3 (0.67–0.1.34)
	1–5 years	21	17	1.64 (1.35–7.78)	0.003^*∗*^	2.19 (1.13–16.22)
	>5 years	42	128	1		1
Job position	Preparation	41	81	0.59 (0.32–1.13)	0.113	0.26 (0.06–1.16)
	Cleaning utensil	5	11	0.66 (0.21–2.15)	0.200	0.81 (0.08–8.48)
	Host students	19	63	1		1
Medical checkups	Yes	1	112	1		1
	No	64	43	0.01 (1.01–1.05)	<0.001^*∗*^	0.12 (1.28–3.16)
Hands washing habit	Yes	64	152	1.26 (0.13–12.37)	0.185	0.26 (0.01–8.98)
	No	1	3	1		1
Finger nail status	Trimmed	54	126	1.13 (0.53–2.43)	0.665	1.56 (021–11.52)
	Not trimmed	11	29	1		1
Wear clean apron	Yes	43	96	1.2 (0.65–1.11)	0.226	0.20 (0.02–2.68)
	No	22	59	1		1
Hands wash with soap	Only with water	12	33	0.84 (0.4–1.75)	0.533	1.68 (0.33–8.53)
	With soap	53	122	1		1
Unpasteurized milk	Yes	15	14	3.02 (1.36–6.71)	0.007^*∗*^	0.19 (1.03–1.42)
	No	50	141	1		1
Eating raw meat	Yes	61	32	5.12 (3.14–15.31)	<0.001^*∗*^	0.03 (1.01–1.16)
	No	4	123	1		1
Eating raw vegetable	Yes	24	63	0.86 (0.47–1.55)	0.386	1.78 (0.48–6.58)
	No	41	92	1		1

AOR = adjusted odds ratio, COR = crude odds ratio.

## Data Availability

All the datasets used to support the findings of this study are available from the corresponding author upon request.

## Abbreviations

AOR: Adjusted odds ratio
AST: Antimicrobial susceptibility testing
ATCC: American type culture collection
CI: Confidence interval
CLSI: Clinical and laboratory standards institute
COR: Adjusted odds ratio
MDR: Multidrug resistance
SPSS: Statistical Package for Social Sciences
TSB: Tryptone soya broth
XLD: Xylose lysine deoxycholate agar
WHO: World Health Organization.
